# PReS-FINAL-1002: Dissecting the dissociation of foxp3 and cd25 expression on cd4+ t cells in synovial fluid identifies three distinct subpopulations of human t regulatory cells present at the chronically inflamed site

**DOI:** 10.1186/1546-0096-11-S2-O2

**Published:** 2013-12-05

**Authors:** D Bending, AM Pesenacker, Q Wu, LR Wedderburn

**Affiliations:** 1 Rheumatology Unit, UCL, Institute of Child Health, London, UK; 2 Imperial College London, London, UK

## Introduction

T regulatory cells (Treg), vital to prevent autoimmunity, are defined by expression of FoxP3 in combination with high CD25 and low CD127 expression. It has been reported, however, that upon *in vitro *activation, conventional T cells (Tconv) can manifest phenotypic marks associated with Treg and, as such, expression of these markers alone is insufficient to determine Treg commitment. A unique feature of Treg is the presence of a Treg specific demethylated region (TSDR) in intron 1 of the *FOXP3 *gene. This distinguishes activated Tconv from *bona fide *Treg. In CD4^+ ^T cells from the joints of children with JIA we have observed a clear dissociation of CD25 and FoxP3 expression. The relationship between CD25, CD127 and FoxP3 expression and commitment to Treg lineage at the inflamed site is unclear; meaningful investigation is hampered by the technical difficulties in isolating cells based on FoxP3 status.

## Objectives

To analyze phenotype and frequency of CD4^+ ^T cells isolated from synovial fluid (SF) from JIA patients based on expression patterns of FoxP3, CD25 and CD127, their *in vivo *turnover, and degree of commitment to the Treg lineage.

## Methods

Peripheral blood mononuclear cells (PBMC) from JIA patients and controls and SF mononuclear cells (SFMC) were analyzed *ex vivo *for the expression of FoxP3, CD25, CD127, Ki67 and PD-1. In addition, SF CD4^+ ^T cells, which were fixed and stained for FoxP3, were sorted in to 4 distinct populations based on CD25, FoxP3 and CD127 expression. DNA was extracted using a modified phenol-based protocol, bisulfite-treated, the TSDR amplified, cloned and sequenced to quantify methylation levels.

## Results

3 populations of CD4^+ ^T cells displaying Treg lineage commitment were identified: Population I (PI), CD4^+^CD127^lo^CD25^lo^FoxP3^hi ^(median of 5.03% of CD4^+ ^T cells in SFMC vs. 0.94% in control, and 1.22% in JIA, PBMC); population II (PII), CD4^+^CD127^lo^CD25^hi^FoxP3^hi^, (median 11.69% CD4^+ ^T cells in SFMC vs. 5.74% in control, and 4.93% in JIA, PBMC); and population III (PIII), CD4^+^CD127^lo^CD25^hi^FoxP3^lo ^(median of 4.13% of CD4^+ ^T cells in SFMC vs. 0.82% in control, and 0.63% in JIA, PBMC); all 3 were significantly enriched compared to controls and displayed low levels of TSDR methylation (median methylation rates: PI, 19.73%; PII, 3.8%; PIII, 15.65%; Tconv, 95.55%). PD-1 expression was higher on all 3 populations when compared to controls, with highest expression on PIII, which also had a lower frequency of Ki67^+ ^cells compared to PI and PII (% ki67^+ ^median: PI, 18.4%; PII, 20.2%; PIII, 8.96%).

## Conclusion

The presence of CD25 and/or FoxP3 alone is insufficient to define Treg populations in the inflamed joint. We propose 3 populations: PI & PII represent Treg expressing high levels of FoxP3 differing in their levels of CD25 but have robust turnover *in vivo*. PIII, with high levels of CD25 but low FoxP3 may represent an exhausted population of Treg (indicated by downregulated FoxP3, high PD-1 and low turnover *in vivo*). Understanding turnover and fate of regulatory cells in JIA will aid definition of how tolerance is lost in this autoimmune disease.

## Disclosure of interest

None declared.

